# Assessing the Effectiveness of *in-situ* Active Warming Combined With Open Top Chambers to Study Plant Responses to Climate Change

**DOI:** 10.3389/fpls.2020.539584

**Published:** 2020-11-20

**Authors:** Esther R. Frei, Luc Schnell, Yann Vitasse, Thomas Wohlgemuth, Barbara Moser

**Affiliations:** ^1^Swiss Federal Institute for Forest, Snow and Landscape Research WSL, Birmensdorf, Switzerland; ^2^Department of Physics, ETH Zurich, Zurich, Switzerland

**Keywords:** air temperature, electric heater (EH), phenology, relative humidity, spatial temperature distribution, warming cables

## Abstract

Temperature manipulation experiments are an effective way for testing plant responses to future climate conditions, especially for predicting shifts in plant phenological events. While passive warming techniques are widely used to elevate temperature in low stature plant communities, active warming has been applied less frequently due to the associated resource requirements. In forest ecosystems, however, active warming is crucial to simulate projected air temperature rises of 3–5 K, especially at the warm (i.e., southern and low elevation) range edges of tree species. Moreover, the warming treatment should be applied to the complete height of the experimental plants, e.g., regenerating trees in the understory. Here, we combined open top chambers (OTCs) with active heat sources, an electric heater (OTC-EH) and warming cables (OTC-WC), and tested the effectiveness of these set-ups to maintain constant temperature differences compared to ambient temperature across 18 m^2^ plots. This chamber size is needed to grow tree saplings in mixture in forest gaps for 3 to 10 years. With passive warming only, an average temperature increase of approx. 0.4 K as compared to ambient conditions was achieved depending on time of the day and weather conditions. In the actively warmed chambers, average warming exceeded ambient temperatures by 2.5 to 2.8 K and was less variable over time. However, active warming also reduced air humidity by about 15%. These results underline the need to complement passive warming with active warming in order to achieve constant temperature differences appropriate for climate change simulations under all weather conditions in large OTCs. Since we observed considerable horizontal and vertical temperature variation within OTCs with temperature differences of up to 16.9 K, it is essential to measure and report within-plot temperature distribution as well as temporal temperature variation. If temperature distributions within large OTCs are well characterized, they may be incorporated in the experimental design helping to identify non-linear or threshold responses to warming.

## Introduction

Climate change affects the structure and function of ecosystems (Walther et al., 2002; Thomas et al., 2004; Parmesan, 2006) by altering growth rates, physiology, survival, and distributions of individuals, populations, species, and communities (Dukes and Mooney, 1999; Hobbie et al., 1999; Morin et al., 2008; Reich et al., 2015; Gruner et al., 2017). Understanding and predicting biological effects of climate change are among the key challenges of current ecological research. Temperature manipulation experiments are an effective way of testing and quantifying plant responses to climate change (Ettinger et al., 2019). They are urgently needed to improve and validate models that predict climate driven shifts in phenological events.

Ecosystem warming experiments can employ passive or active warming methods and the efficiency of the different methods has been reviewed several times (e.g., Shaver et al., 2000; Rustad et al., 2001; Aronson and McNulty, 2009; Wolkovich et al., 2012; De Frenne, 2015; Ettinger et al., 2019). Passive warming methods include greenhouses or open top chambers (OTCs) that elevate daytime temperatures via a greenhouse-effect by transmitting solar radiation and trapping the heat within the chamber (Kennedy, 1995; Marion et al., 1997) or IR reflective curtains that increase night-time soil temperatures by reducing the infrared radiation heat loss from vegetation and soil surface at night (Beier et al., 2004; Emmett et al., 2004). Warming chambers for ecosystem manipulation studies consist of variously sized and shaped greenhouses, tents and OTCs. The latter were initially developed for gas exchange experiments. The passive warming of these chambers was an unintended side effect making them interesting for climate warming research (e.g., Drake et al., 1989). Due to their low infrastructure, maintenance and budget requirements OTCs have been widely used to elevate temperature in open low stature plant communities, such as remote arctic and alpine tundra ecosystems (e.g., Elmendorf et al., 2012), grassland steppe (Klein et al., 2005), temperate grasslands (Carlyle et al., 2011) and saltmarshes (Gedan and Bertness, 2009), but they were rarely used in taller-stature plant communities (Welshofer et al., 2018). OTCs are ineffective without solar irradiance, and thus have only limited potential for applications in forest ecosystems (De Frenne et al., 2010).

Active warming methods employ an external heat source (e.g., Cleland et al., 2006; Kimball et al., 2008; Dawes et al., 2011). They are independent of solar radiation and warming is implemented by either constant energy output (wattage), or a heating system with a feedback control system, which maintains a constant temperature difference between warmed and control treatments (Ettinger et al., 2019). The extensive associated energy demands (e.g., Pelini et al., 2011) as well as infrastructure and maintenance requirements limit the circumstances under which active warming techniques can be applied (Aronson and McNulty, 2009), such as their implementation in natural forest ecosystems.

IR heaters are the most frequently used external heat source for active warming of short-stature plant communities (e.g., Harte and Shaw, 1995; Luo et al., 2001; Shaw et al., 2002; Kimball et al., 2008), but they have recently also been used for the heating of a tropical forest understory (Kimball et al., 2018; Wood et al., 2019). Another valuable, often used active warming field method are soil warming cables (e.g., Peterjohn et al., 1993; Rustad et al., 2001; Melillo et al., 2017). However, both methods have difficulties in achieving target temperatures under unfavorable weather conditions, such as strong winds or rainstorms (Ettinger et al., 2019).

Only few climate manipulation experiments achieve warming with electric free air heaters that are combined with OTCs. Yet, such actively warmed chambers can provide a more accurate quantitative temperature control and have the potential to simulate non-linear and threshold responses to warming (Jentsch et al., 2007; Amthor et al., 2010). Norby et al. (1997) established an air-warming method by using a fully regulated active open-top chamber, which was heated by passing warmed air through the chamber. Bronson et al. (2008) built larger actively warmed chambers to study the effects of elevated temperatures on boreal black spruce (*Picea mariana*) and combined the air warming approach with soil-warming cables. In the framework of the whole ecosystem experiment SPRUCE (Spruce and Peatland Response Under Climatic and Environmental Change) a promising system of new active air warming chambers has been developed that can achieve multiple levels of experimental warming in combination with deep soil warming in a black spruce-*Sphagnum* peat bog in northern Minnesota (Barbier et al., 2013; Hanson et al., 2017; Richardson et al., 2018). Another whole ecosystem experiment, the Climate Change Experiment (CLIMEX), used fully enclosed air warming chambers consisting of large-scale greenhouses that enclosed an existing intact boreal forest ecosystem with mature trees and shrubs (Beerling, 1999). Medhurst et al. (2006) implemented a series of experiments using fully enclosed whole-tree chambers with air warming of single adult boreal spruce trees (*Picea abies*) in Norway. The same system has thereafter been deployed for *Eucalyptus* studies in Australia (Barton et al., 2010) and has subsequently been adapted to improve temperature control system (Crous et al., 2013; Drake et al., 2019).

Ettinger et al. (2019) reviewed 17 studies that applied active warming methods and included either multiple levels of warming or precipitation treatments. Their meta-analysis showed that warming treatments were confounded with a suite of indirect and feedback effects that are likely to affect biological responses studied in climate change experiments such as growth or phenology. They also underline the importance of considering spatial and temporal variation in plot temperatures. However, the study of Ettinger et al. (2019) only addressed among-plot variation while a better understanding of within-plot temperature variation, which may have similar ecological implications, would be equally important.

In this study, we aimed at developing an infrastructure suitable to warm the canopy of young temperate forest communities, i.e., 3- to 10-year-old tree seedlings growing in forest gaps which can be easily and relatively autonomously be deployed in the field. We also wanted to assess the feasibility of a warming experiment with a large number of tree saplings in the field. We combined OTCs with two different active heat sources, an electric heater (hereafter referred to as “OTC-EH”) and warming cables (hereafter “OTC-WC”), and tested the effectiveness of these set-ups to maintain a constant temperature difference of 3 K above ambient temperature across an 18 m^2^ plot (5.2 m maximum basal diameter, 2.1 m OTC panel height). We measured air temperature and humidity distribution in the passively warmed OTC plot (hereafter “OTC-CTRL”), as well as in the two actively warmed OTC plots (OTC-EH and OTC-WC) and determined the achieved warming and humidity changes relative to ambient conditions (hereafter “Full-CTRL”) in order to test the efficiency of the different set-ups. Testing empty OTCs allowed us to focus on physical effects while minimizing biotic disturbances. Spatial and temporal temperature variation within plots and among treatments was analyzed based on more than 180,000 temperature records. Specifically, we asked the following questions: (1) How much warming can be achieved with (a) an OTC (passive warming only) and (b) an OTC combined with two different heat sources (active warming)? (2) Are the achieved warming effects constant over time? (3) If the achieved warming is not constant, what environmental factors cause variation in the effects? (4) Is the temperature evenly distributed across plots?

## Materials and Methods

### Experimental Design

**FIGURE 1:**
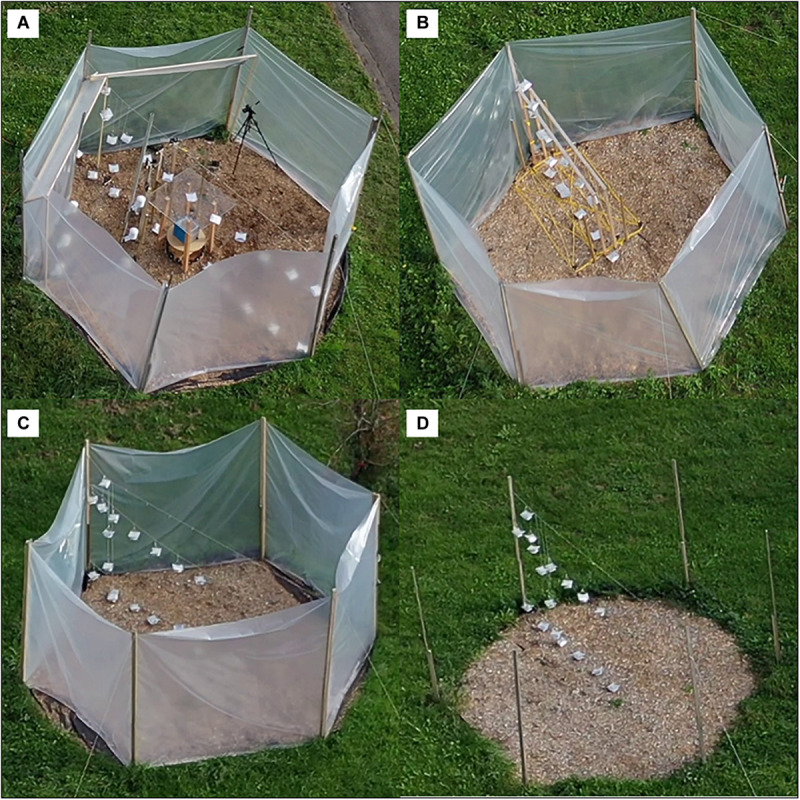
Aerial view of experimental OTC-EH plot 1 **(A)**, OTC-WC plot 2 **(B)**, OTC-CTRL plot 3 **(C)**, and Full-CTRL plot 4 **(D)**.

Four experimental plots were installed on a grassland in the outdoor area of the research facility WSL in Birmensdorf, Switzerland (47.3616°N, 8.4556°E, 560 m a.s.l.), in August 2019. The four hexagonal plots had a maximum basal diameter of 5.2 m and a height of 2.1 m. The six corners were marked with roof battens stacked into the soil. Three of the plots were framed with a 2.1 m × 16 m plastic foil (180 μm thick PP foil and 200 μm thick Lumisol clear AF) forming a tall OTC, whereas the fourth plot served as full control. The grass-covered soil surface of the plots was covered with a water-permeable lining and a 0.05 m thick layer of wood chips to prevent interference from transpiring plants. The first plot was experimentally warmed with an electric heater (plot 1, OTC-EH; Figure 1A), the second plot contained electrical resistance-heating cables laid out on the ground surface (plot 2, OTC-WC; Figure 1B), the third plot was passively warmed through the greenhouse effect of the OTC (plot 3, OTC-CTRL; Figure 1C) and the fourth plot was a full control plot without OTC, in which only the ground was covered with the water-permeable lining and wood chips (plot 4, Full-CTRL; Figure 1D).

**FIGURE 2:**
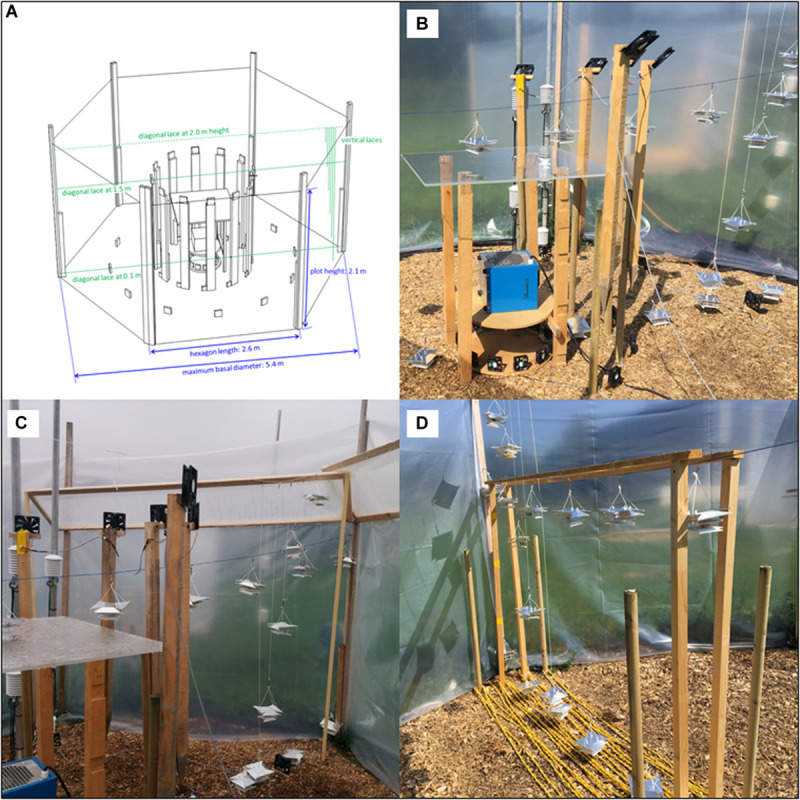
Schematic view of OTC-EH plot 1 with full-plot set-up **(A)** and side-views of OTC-EH plot 1 with different set-ups **(B,C)** and OTC-WC plot 2 **(D)**.

The OTC-EH plot 1 was equipped with a 3-kW electric heater (TROTEC TDE25) located in the center of the plot and protected by a 100 cm × 100 cm transparent acrylic glass plate at 115 cm height. Ten large ventilators (DC 119 × 119 × 25 mm 24 V 195 m^3^/h, NMB) and 17 small ventilators (DC 80 × 80 × 25 mm 12 V 70 m^3^/h) were arranged in the Eastern half of the plot around the electric heater in order to distribute the warmed air within the OTC (Figures 2A,B). For the last experimental run (see section “Experimental Runs”), the whole plot 1 was equipped by 24 large and 24 small ventilators and wall-shields creating a 30° frustum to direct the warm air toward the ground (Figures 2A,C). In OTC-WC plot 2, 100 m soil heating cable (CAMPLEX, 11 W/m) was laid out as double-string and in spirals on the ground surface with 20 or 40 cm distance between neighboring cables, covering an area of a total of 6 to 9 m^2^ in the Eastern half of the plot (Figure 2D). OTC-CTRL plot 3 was not equipped with any heating device to test passive warming effects of the OTC. Full-CTRL plot 4 was only equipped with the six corner roof battens and the permeable lining with the wood chips layer on the ground that allowed for identical plot temperature measurements as in plots 1–3 (for material details in Supplementary Table S1).

### Temperature and Humidity Measurements

We measured air temperatures and humidity in each plot with 68 iButton loggers (36 DS 1922L Thermochron loggers, which measured only temperature and 32 DS 1923 Hygrochron loggers, which measured temperature and air humidity, Maxim Integrated, San Jose, CA, United States). To determine spatial temperature and humidity variation within each plot, two strings were spanned diagonally across each plot at 0.1 m and 1.5 m height. Six iButton loggers were attached to each diagonal string in distances of 0.5 m from the plot center to the Eastern plot edge. Additionally, five vertically spanned strings of different lengths (0, 0.5, 1.0, 1.5, and 2.0 m) were attached to a third diagonal string at 2.0 m height in a distance of 1.8 m from the plot center to the Eastern plot edge. Five iButton loggers were attached to these vertical strings. Each iButton logger was protected from direct solar radiation with a custom-fabricated radiation shield (referred to as “Small-Radshield” in Terando et al., 2017). We showed in a preliminary test under open field conditions that this type of radiation shield was more efficient in reducing heating up of the temperature sensors by solar radiation than the radiation shield described by Holden et al. (2013) or a manufacturer-recommended protective cover for outdoor temperature transmitters (TFA Dostmann GmbH & Co. KG). The iButtons protected with the small radiation shields most closely followed the temperatures measured at the climate station of the Model Ecosystem Facility/MODOEK nearby, an ATMOS 41 All-in-One Weather station from METER Group (Supplementary Figure S1). Even though the iButtons recorded temperatures approximately 0.5°C above the climate station during nighttime and 2°C during daytime, this does not affect absolute differences between individual iButtons (temperature resolution 0.0625°C) since they were all covered by the same type of radiation shield. The iButton loggers were set to record temperature and air humidity every 10 min.

### Experimental Runs

Six experimental runs were conducted between August 23rd and December 6th 2019 featuring four slightly different set-ups. During the runs the effects of the arrangement of ventilators in the OTC-EH plot and the spacing of warming cables in the OTC-WC plot was tested (for details Table 1).

**TABLE 1:** Details of the six experimental runs conducted from August to December 2019, including their duration as well as the arrangement of the five ventilators at 170 cm height in OTC-EC and the warming cables in OTC-WC.

**Run**	**OTC-EH Ventilator arrangement**	**OTC-WC Distance between warming cables**	**Start date**	**End date**	**Duration [hours]**
1	5 ventilators at 170 cm above ground blowing toward the ground at a 30° angle, no frustum (Figure 1A)	20 cm	August 23	August 26	60
2	Same as run 1	40 cm	September 5	September 18	330
3	Same as run 1	40 cm	October 10	October 15	105
4	5 ventilators at 170 cm height blowing horizontally toward a 30° frustum designed to direct the warm air toward the ground (Figure 1B)	40 cm	October 18	October 21	60
5	Same as run 4	40 cm	October 26	November 1	138
6	12 ventilators at 170 cm height, blowing horizontally toward a 30° frustum designed to direct the warm air toward the ground, whole plot ventilated	40 cm	December 2	December 6	72

### Data Analysis

All statistical analyses were conducted using R version 3.6.1 (R Core Team, 2019). We first calculated means, standard deviations, and ranges (=differences between highest and lowest values) of all logger temperatures in each plot. Based on these, we calculated the temperature differences TD_Treatment_ = T_Treatment_−T_Full–CTRL_ between each treatment and the Full-CTRL. As an aggregated measure of the temperature increase achieved in the treatments with respect to ambient temperature, we determined the mean temperature difference (hereafter referred to as meanTD) between each treatment and Full-CTRL for each experimental run as $\mathrm{meanTD}=\frac{1}{n}\,\sum_{i=1}^{n}{\text{TD}}_{\mathrm{Treatment},i}$. The meanTD between OTC-CTRL and Full-CTRL describes the passive warming effect of the OTC (without additional heating), whereas the meanTDs between the actively heated plots and Full-CTRL reveal the total achieved warming, which comprises the combination of passive and active warming effects. Similarly, we calculated the mean difference in relative humidity between treatments and Full-CTRL (meanRHD).

Student’s *t*-tests (Welch Two Sample *t*-tests) were used to check if temperature differences between treatment and full control differed between day and night. Resulting *t*-values were Bonferroni corrected for multiple comparisons. To test whether the effectiveness of the warming treatments depended on weather conditions, we used linear mixed effects models and multi-model inference (Burnham and Anderson, 2002). The hourly average temperature differences between treatments (OTC-EH, OTC-WC, OTC-CTRL) and Full-CTRL were related to the following predictors: time of the day (day or night), hourly mean air temperature, rain (yes or no), and hourly mean wind speed (log transformed). Relative humidity and global radiation were not included in the models because they were highly correlated with air temperature. Because time of the day significantly interacted with temperature, rain and wind speed in an overall model, we decided to fit separate models for day (from sunrise to sunset) and night (from sunset to sunrise). Predictor and response variables were aggregated to hourly values. Linear mixed effect models were calculated with the lme-function in package nlme (Pinheiro et al., 2020). The identifier of the experimental run was included as random effect. Continuous predictor variables were standardized to a mean of zero and unit standard deviation using the function decostand from the package vegan (Oksanen et al., 2019). Automatic backward model selection using the function dredge (package MuMIn; Bartoń, 2019) was applied to find the simplest model explaining the highest proportion of variation. We report all models with a ΔAICc < 2 from the best fitting model. The times of sunrise and sunset for each day at the experiment location were determined using the R package suncalc (Thieurmel and Elmarhraoui, 2019). Weather parameters from two climate stations located within a distance of 100 m from our experimental plots were used: the LWF Uitikon Freiland station belonging to the Swiss Long-term Forest Ecosystem Research program LWF^1^, which is part of the UNECE Co-operative Program on Assessment and Monitoring of Air Pollution Effects on Forests ICP Forests^2^ (Schaub et al., 2011): LWF Uitikon Freiland station (for precipitation and global radiation), and the Model Ecosystem Facility/MODOEK, WSL climate station, an ATMOS 41 All-in-One Weather station from METER Group (for air temperature and wind speed).

As measures for spatial variability of temperature within each experimental plot, the mean within-plot temperature standard deviation for each experimental run (hereafter referred to as MTSD) were calculated as $\mathrm{MTSD}=\frac{1}{n}\,\sum_{i=1}^{n}\sigma \,\left(T_{\mathrm{Treatment},i}\right)$. We also determined mean and maximum range of plot temperatures for each experimental run (hereafter referred to as meanTR and maxTR). Similarly, we calculated the mean within-plot standard deviation of relative humidity (MRHSD). We calculated radial temperature gradients at 10 cm height from the center toward the outer edge of the experimental plot as the difference between the temperatures measured at 250 and 50 cm from the center divided by the horizontal distance between the two logger positions and reported the mean gradients for each experimental run (hereafter referred to as mean gradient).

## Results

### Passive and Active Warming Effects

**FIGURE 3:**
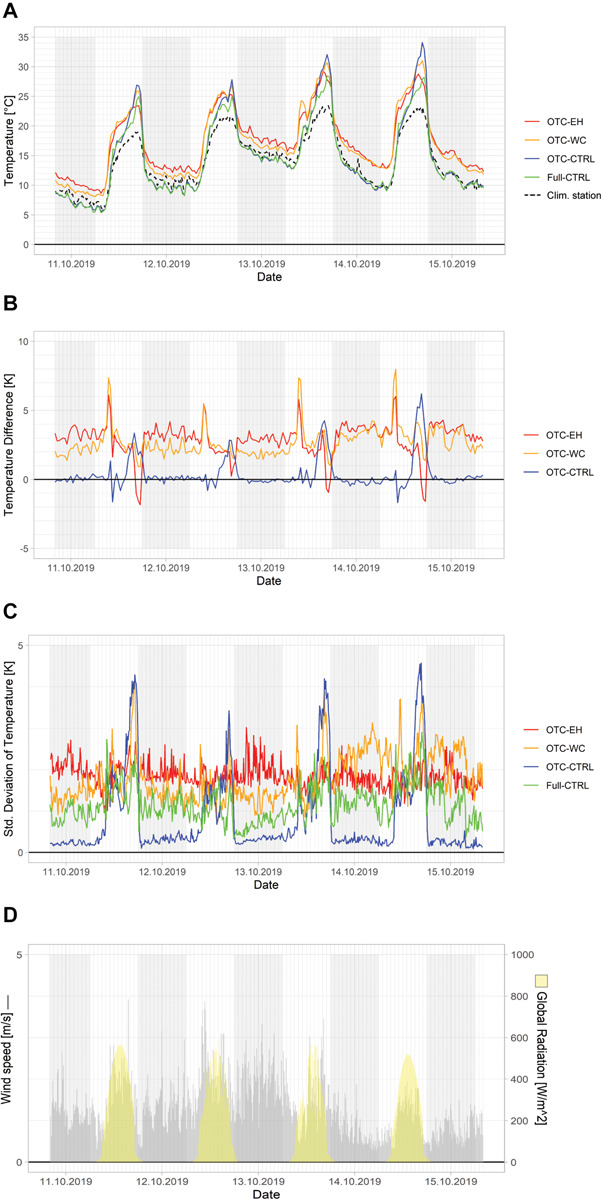
Temperature measurements during experimental run 3 (October 10–15, 2019): **(A)** Mean absolute plot temperatures (Clim. Station: air temperature measured at the MODOEK climate station), **(B)** temperature differences with respect to Full-CTRL, **(C)** standard deviations of within plot temperatures, and **(D)** average wind speed and global radiation measured at the climate stations. The gray bars indicate wind speed and the yellow bars global radiation from climate stations. Gray shading indicates the time intervals between 6 pm and 6 am (not actual dark hours).

Average plot temperatures, i.e., the mean temperatures of all individual temperature logger measurements within a plot at a given point in time, were mostly higher in the two actively warmed plots (OTC-EC and OTC-WC) than in the passively warmed plot (OTC-CTRL) and the full control plot (Full-CTRL; Figure 3). Determining passive and active warming effects turned out to be challenging during sunrise and sunset: OTC-EC and OTC-WC were located approximately 10 m to the west of OTC-CTRL and Full-CTRL, which means that the actively heated plots were hit by direct sunlight earlier than the control plots in the morning. The opposite effect occurred during sunset in the evening. Consequently, pairwise temperature difference between the heated plots and Full-CTRL peaked for a short period of time in the early morning (Figure 3B), while temperatures in OTC-CTRL and Full-CTRL were higher than in the actively warmed plots around sunset (Figure 3A) seemingly leading to lower heating effect with respect to Full-CTRL in actively heated plots than in OTC-CTRL (Figure 3B).

Nighttime temperatures in the actively warmed OTCs were elevated in comparison to OTC-CTRL and Full-CTRL. Average plot temperature of the passively warmed OTC-CTRL showed a different pattern than the actively warmed plots: during the night, the temperatures in OTC-CTRL were similar to those in Full-CTRL, whereas during daytime the former warmed more rapidly than the latter, as shown by the increasing temperature difference between OTC-CTRL and Full-CTRL (Figure 3). We also found seasonal variation in the achieved passive warming: While temperatures in OTC-CTRL reached the temperatures of the actively warmed plots for part of the day in August and September, the achieved temperature difference became smaller toward winter because of the shorter day length and the lower elevation angle of the sun (Table 2).

**TABLE 2:** Mean temperature differences (meanTD) between treatments and Full-CTRL for the six experimental runs.

**Parameter**	**Plot**	**Experimental run**						**All runs**		
		**1**	**2**	**3**	**4**	**5**	**6**	**Average**	**Min**	**Max**
meanTD [K]	OTC-EH vs. Full-CTRL	2.79	2.58	2.91	2.96	2.88	3.02	2.86	2.58	3.02
	OTC-WC vs. Full-CTRL	3.57	2.31	2.72	2.14	1.99	2.35	2.51	1.99	3.57
	OTC-CTRL vs. Full-CTRL	0.94	0.55	0.39	0.18	0.24	0.09	0.40	0.09	0.94

The average warming measured as mean temperature difference (meanTD) relative to Full-CTRL (mean difference between warmed plot and full control) in the six experimental runs between August and December 2019 ranged from 2.58 to 3.02 K with the electric heater (OTC-EH), from 1.99 to 3.57 K with the warming cables (OTC-WC), and from 0.09 to 0.94 K with passive warming only (OTC-CTRL; Table 2).

### Weather Effects on Achieved Warming During Day and Night

The meanTD of OTC-EH and Full-CTRL was 0.88 K larger during the night than during the day (3.25 K vs. 2.37 K, *t* = 31.46, *P* < 0.001). During the day, the meanTD was primarily affected by rain events and air temperature: the warming effect was generally greater during dry than rainy weather (Table 3A) and when ambient temperatures were lower. Higher wind speed also resulted in larger temperature differences, i.e., more warming, although the effect was smaller than that of rain and air temperature. At night, weather acted again as the main driver of temperature differences, with smaller differences during rain events. The effects of temperature and wind speed, by contrast, were inverse to their effects during the day: While higher ambient temperatures increased temperature differences, high wind speed led to smaller warming effects.

**TABLE 3:** Model selection results for differences between treatments and Full-CTRL separately for day (defined as time from sunrise to sunset) and night (defined as time from sunset to sunrise).

	**Top LMM**	**Explanatory variable**	**Linear mixed model**			**Multi-model inference**	
			**Estimate**	**SE**	***P***	**df**	**AICc**
(A) Temperature difference between OTC-EH and Full-CTRL							

*Day*							

	1	rain	−0.47	0.20	0.020	343	994.24
		air temperature	−0.29	0.07	<0.0001		
		wind speed	0.11	0.05	0.035		

*Night*							

	1	rain	−0.60	0.08	<0.0001	414	559.97
		air temperature	0.42	0.05	<0.0001		
		wind speed	−0.29	0.02	<0.0001		

(B) Temperature difference between OTC-WC and Full-CTRL							

*Day*							

	1	rain	−0.66	0.18	0.0003	343	925.12
		air temperature	0.15	0.07	0.027		
		wind speed	−0.09	0.05	0.054		
	2	rain	−0.69	0.18	<0.0001	344	926.74
		air temperature	0.15	0.07	<0.0001		

*Night*							

	1	wind speed	−0.58	0.03	<0.0001	414	609.94
		rain	−0.49	0.08	<0.0001		
		air temperature	0.19	0.05	<0.0001		

(C) Temperature difference between OTC-CTRL and Full-CTRL							

*Day*							

	1	air temperature	0.84	0.07	<0.0001	344	1009.26
		wind speed	0.11	0.05	0.044		
	2	air temperature	0.87	0.08	<0.0001	343	1010.91
		rain	0.15	0.21	0.469		
		wind speed	0.11	0.06	0.051		

*Night*							

	1	wind speed	−0.04	0.01	<0.0001	415	−302.44
		air temperature	0.03	0.01	0.030		

*The full model included the following explanatory variables: air temperature (continuous), presence of rainfall (binomial), and average wind speed (continuous, log-transformed). Experimental run was included in the models as random effect. Starting with the full model, multi-model inference was used to select the simplest models explaining the highest proportion of variation. The top linear mixed models with ΔAICc < 2 are shown (*N*_*Day*_ = 352, *N*_*Night*_ = 423).*

The meanTD of OTC-WC and Full-CTRL was 0.21 K larger during the night than during the day (2.48 K vs. 2.28 K, *t* = 6.71, *P* < 0.001). During the day, two models rendered almost equal goodness of fit (ΔAICc < 2). In both models, temperature differences, i.e., the amount of warming, was negatively affected by rainy weather, while air temperatures showed a much smaller but positive effect (estimate of −0.6 for rain and 0.1 for temperature, respectively; Table 3B). Although wind speed was included in the top model, it did not add much explanatory power (ΔAICc = 1.6). At night, rainy conditions considerably decreased warming effects and higher air temperature again resulted in larger temperature differences. In contrast to daytime, wind speed resulted as the most important factor during the night, significantly reducing warming effects.

For OTC-CTRL, the meanTD was 0.58 K smaller during the night than during the day (0.09 K vs. 0.67 K, *t* = −23.70, *P* < 0.001). During daytime, temperature differences increased first and foremost with air temperature (Table 3C) and to a lower degree with higher wind speeds. Interestingly, rain events were negligible (included in model 2 but not significant). At night, higher air temperatures increased temperature differences, whereas higher wind speeds reduced them.

### Spatial Temperature Variation

Temperature substantially varied within the plots. We found greatest spatial temperature variation in OTC-EH and OTC-WC with average standard deviations of within-plot temperatures (MTSD) of 1.64 to 3.50 K and 1.64 to 2.84 K, respectively (Table 4). The corresponding mean range between the highest and the lowest temperature (meanTR) in these plots amounted from 4.64 to 9.65 K and 4.59 to 7.42 K, respectively, with greatest differences (maxTR) reaching up to 10.98 and 16.93 K. In Full-CTRL the spatial temperature varied about half as much as in the actively warmed plots with MTSDs of 0.35 to 1.38 K and meanTR of 0.95 to 3.64 K. The maxTR observed in Full-CTRL was 11.21 K. Overall, the spatial temperature variation was smallest in OTC-CTRL with MTSDs of 0.23 to 0.88 K, meanTR of 0.71 to 2.70 K, and maxTR of 5.62 to 15.51 K. The mean radial temperature gradients at ground level (i.e., the temperature gradient from the plot center to the plot wall) in OTC-EH ranged between +4.43 and +1.56 K/m. In the other plots, much smaller gradients were observed with +0.81 to −0.12 K/m in OTC-WC, +0.17 to −0.60 K/m in OTC-CTRL, and +0.28 to −0.05 K/m in Full-CTRL.

**TABLE 4:** Within plot spatial temperature variation as characterized by the mean standard deviation (MTSD), mean (meanTR) and maximum (maxTR) temperature range of all loggers in a plot.

**Parameter**	**Plot**	**Experimental run**						**All runs**		
		**1**	**2**	**3**	**4**	**5**	**6**	**Average**	**Min**	**Max**
MTSD [K]	OTC-EH	1.88	1.83	1.85	1.64	1.74	3.50	2.07	1.64	3.50
	OTC-WC	2.84	1.64	1.80	1.72	1.67	2.34	2.00	1.64	2.84
	OTC-CTRL	0.88	0.69	0.82	0.26	0.40	0.23	0.55	0.23	0.88
	Full-CTRL	1.38	1.09	1.15	0.40	0.50	0.35	0.81	0.35	1.38
meanTR [K]	OTC-EH	5.93	5.90	5.68	4.64	5.17	9.65	6.16	4.64	9.65
	OTC-WC	7.42	4.59	5.04	4.84	4.86	6.69	5.57	4.59	7.42
	OTC-CTRL	2.70	2.00	2.44	0.84	1.15	0.71	1.64	0.71	2.70
	Full-CTRL	3.64	2.88	2.94	1.12	1.40	0.95	2.15	0.95	3.64
maxTR [K]	OTC-EH	9.23	10.98	9.16	8.68	10.97	10.65	9.95	8.68	10.98
	OTC-WC	15.54	16.93	12.37	7.63	8.59	13.79	12.48	7.63	16.93
	OTC-CTRL	14.62	15.51	14.55	6.62	13.54	5.62	11.74	5.62	15.51
	Full-CTRL	11.21	10.71	9.86	7.90	8.79	5.95	9.07	5.95	11.21
mean temperature gradient [K/m]	OTC-EH	2.23	2.25	2.18	1.56	1.99	4.43	2.44	1.56	4.43
	OTC-WC	0.81	−0.07	0.01	−0.12	0.03	0.77	0.24	−0.12	0.81
	OTC-CTRL	0.04	0.17	−0.60	−0.14	−0.10	0.11	−0.09	−0.60	0.17
	Full-CTRL	0.12	0.23	0.28	−0.05	0.01	0.06	0.11	−0.05	0.28

*Values are indicated for the individual experimental runs and summarized over all runs. For the mean radial temperature gradient from the center to the edge of the plot only measurements at 10 cm height were considered.*

In the actively warmed plots, temperatures were 2.28 and 2.46 K higher (OTC-EH and OTC-WC, respectively) at 10 cm as compared to 150 cm above ground. In the two plots without active warming (i.e., OTC-CTRL and Full-CTRL), however, vertical temperature differences were small and temperatures at 10 cm were slightly lower than at 150 cm above ground (OTC-CTRL: ΔT = −0.18 K; Full-CTRL: ΔT = −0.52 K; Table 5).

**TABLE 5:** Mean temperatures, standard deviation of temperatures and differences between mean temperatures at 10 and 150 cm over all six experiment runs.

**Plot**	**Height above ground [cm]**	**Mean temperature [°C]**	**Standard deviation [K]**	**ΔT [K]**
OTC-EH	10	17.45	6.59	2.28
	150	15.17	7.36	
OTC-WC	10	16.75	7.45	2.46
	150	14.29	7.99	
OTC-CTRL	10	13.08	7.94	−0.18
	150	13.26	7.12	
Full-CTRL	10	13.21	7.78	−0.52
	150	13.73	8.22	

### Air Humidity Variation Between and Within Treatments

Relative air humidity in the actively warmed plots (OTC-EH and OTC-WC) was on average 13.4% lower than ambient humidity (Table 6), whereas in OTC-CTRL, air humidity was only reduced by 2.1% on average (Table 6). Relative humidity varied considerably in actively warmed plots with average within-plot standard deviations of 7.7 and 10.0% in contrast to the two control plots with average within-plot standard deviations of only 2.5 and 2.2 % (Table 7).

**TABLE 6:** Mean differences in relative humidity (meanRHD) between treatments and Full-CTRL for the six experimental runs.

**Parameter**	**Plot**	**Experimental run**						**All runs**		
		**1**	**2**	**3**	**4**	**5**	**6**	**Average**	**Min**	**Max**
meanRHD [%]	OTC-EH vs. Full-CTRL	−10.98	−10.13	−12.69	−15.26	−18.47	−12.69	−13.37	−18.47	−10.13
	OTC-WC vs. Full-CTRL	−15.79	−10.63	−13.81	−12.98	−13.49	−13.75	−13.41	−15.79	−10.63
	OTC-CTRL vs. Full-CTRL	−3.44	−3.36	−1.17	−0.78	−2.36	−1.44	−2.09	−3.44	−0.78

**TABLE 7:** Spatial variability of relative humidity within plots measured as mean standard deviation of relative humidity (MRHSD) for the six experimental runs.

**Parameter**	**Plot**	**Experimental run**						**All runs**		
		**1**	**2**	**3**	**4**	**5**	**6**	**Average**	**Min**	**Max**
meanRHSD [%]	OTC-EH	6.59	6.07	7.23	9.51	9.69	7.23	7.72	6.07	9.69
	OTC-WC	12.61	6.89	9.39	10.72	9.47	11.03	10.02	6.89	12.61
	OTC-CTRL	3.04	2.55	3.55	2.52	2.05	1.34	2.51	1.34	3.55
	Full-CTRL	2.90	2.94	3.06	1.44	1.52	1.50	2.23	1.44	3.06

## Discussion

Our findings show that complementing large OTCs with active warming equipment resulted in an average achieved warming of 2.5 to 2.9 K and that the warming effect varied over time. It was strongly reduced by rain and also partly influenced by air temperature and wind. Substantial horizontal and vertical within-chamber temperature variation was observed. Relative air humidity in general showed opposite patterns compared to temperature changes.

### Passive and Active Warming Effects

The average warming achieved in the six experimental runs between August and December 2019 was around six to seven times higher in the actively warmed plots with an electric heater (meanTD = 2.86 K) or warming cables (meanTD = 2.51 K) as external heat source than in the passively warmed OTC-CTRL (meanTD = 0.40 K; Table 2). One of the main reasons explaining this difference is that passive warming was only effective during daylight hours when the plots were warmed by solar irradiance (meanTD_*Day*_ = 0.67 K). Therefore, passive warming in OTC-CTRL was obviously negligible at night (meanTD_*Night*_ = 0.09 K; Figure 3). The greatest passive warming effect (maxTD between OTC-CTRL and Full control) was reached around 4 pm. It therefore lagged 2.5–3 h behind the peak solar irradiance, which occurred around 1 pm CEST (Figure 3D). This is the same time of the day when also ambient air temperature reached its maximum (dashed line in Figure 3A). The passive warming effect is due to “trapping” of infrared radiation emitted by the ground surface inside the OTC (greenhouse effect). In particular, since the degree of passive warming is correlated with the ground surface temperature and it takes some time to heat the ground, air temperature lags behind solar irradiance. Other studies with OTCs at a High Arctic site as well as at a site in Montreal confirmed that warming occurred mainly during day-time hours (Marion et al., 1997; Dabros et al., 2010) and that warming correlated positively with solar irradiance at the site (Bokhorst et al., 2013). In contrast, active warming can be effective 24 h a day and we found an even greater warming effect at night (OTC-EH: meanTD_*Day*_ = 2.37 K vs. meanTD_*Night*_ = 3.25; OTC-WC: meanTD_*Day*_ = 2.28 K vs. meanTD_*Night*_ = 2.48 K). We suggest that the active warming is more effective because of increased convection effects at night that support efficient air mixing inside the OTCs.

Our results demonstrate that topography and the relative positioning of the reference temperature measurement are important shortly after sunrise and before sunset, particularly in fall when solar elevation above the horizon is low. This resulted in short periods of time when seemingly erratic temperature differences between the two actively warmed OTCs (OTC-EH, OTC-WC) and Full-CTRL were measured (Figure 3B), which were due to earlier sunrise and earlier sunset in actively warmed OTCs as compared to OTC-CTRL and Full-CTRL. Topography and the location of the reference temperature measurement should be evaluated carefully when designing a warming experiment with large OTCs that are potentially scattered over an area of several hundred square meters, particularly if the degree of warming is regulated relative to the control. Other sources of confounding variation might include shading by the OTC construction or other technical equipment.

### Environmental Influences on Warming

The mixed model analysis revealed that air temperature had the greatest influence on passive warming (OTC-CTRL) during the day with higher air temperatures resulting in greater warming. A likely explanation for this finding is that air temperature is related to weather conditions: higher temperatures are commonly observed during sunny days when high solar irradiance leads to efficient passive warming of the OTC. Interestingly, rain events did not significantly influence passive warming. However, the effect of rain should be interpreted with caution because there were only very short periods of rainfall during the experiments and during some of the experimental runs rainfall was completely absent. At night, when the warming in OTC-CTRL was nearly zero, the influence of the considered weather factors was only weak (Table 3).

The active warming techniques, however, were most importantly influenced by rain, which reduced the effectiveness of active warming by roughly 0.6 K both during day and night. Reduced warming during rainstorms was also observed in an experiment using soil warming cables buried at 10 cm depth (Peterjohn et al., 1993). This suggests that additional heating power is required to maintain constant temperature differences during rain events. Higher wind speed consistently reduced temperature differences in all treatments at night and in OTC-WC also during the day, which is likely due to more efficient mixing of air inside the OTCs with ambient air reducing the warming effect. Numerical simulations showed that the wind-induced mixing effects inside an OTC become stronger with increasing wall heights (Cunningham et al., 2013). Warming effects increased with higher wind speeds during the day in OTC-EH and OTC-CTRL because strong wind cools ambient air in contrast to less circulated air within OTCs, which is warmed by solar irradiance during the day.

Environmental influences and temporal temperature variation might be reduced by installing additional heating combined with a temperature feedback system that controls the power output of the heaters. Temperature variation in such feedback controlled systems has been found to be smaller than in set-ups with constant wattage heating (Ettinger et al., 2019).

### Warming Effects on Air Humidity

Warming the air by 2.5 K would lead to approximately 12.5% higher potential evapotranspiration (Dabros et al., 2010) and consequently to reduced soil moisture and air humidity, which may cause drought stress for plants (Amthor et al., 2010). In our experiment, we observed that relative humidity in OTC-EH and OTC-WC was 13.4% lower than in Full-CTRL on average, whereas in OTC-CTRL relative humidity was reduced by only 2.1% (Table 6). Similar humidity reductions as in our active warming treatments were observed in the SPRUCE whole ecosystem warming experiment with large OTCs in northern Minnesota (Hanson et al., 2017). According to climate change scenarios, there may be regional and temporal variation in the extent and direction of predicted air humidity changes (Stocker et al., 2013). Thus, several experiments compensated for reduced air humidity by moisturizing the heated air with humidification systems (e.g., Thompson et al., 1992). However, such installations are resource intensive and technically challenging. As an increase of air temperature is naturally associated with a reduction in relative humidity, we believe that both effects should be included in climate change experiments. Since not only temperature but also humidity changes may influence plant physiology, growth and phenology as well as soil microbiology, climate change experiments should not only measure and report air temperature but also soil temperature and moisture as well as air humidity (Amthor et al., 2010; Ettinger et al., 2019).

### Spatial Temperature Variation

Our temperature measurements in the full control plot showed that the horizontal and vertical temperature distribution under ambient conditions was relatively homogeneous within the volume of our OTCs (5.2 m diameter, 2.1 m height). The MTSD in this plot was 0.81 K and the meanTR was 2.15 K (Table 4). Temperature varied even less in OTC-CTRL with MTSD = 0.55 K and a meanTR = 1.64 K (Table 4), which suggests that the OTC had an equalizing effect on within-chamber temperatures. We propose that the OTC walls acted as temperature-equalizing elements, which warmed or cooled all air layers within the OTC similarly whereas the ground affected air warming or cooling only within a few centimeters.

In the actively warmed plots (OTC-EH and OTC-WC), spatial temperature variation was substantially greater than in OTC-CTRL and Full-CTRL with MTSDs of 2.07 and 2.00 K and meanTRs of 6.16 and 5.57 K, respectively (Table 4). The overall larger temperature variation in the actively warmed plots as compared to OTC-CTRL can, at least partly, be explained by vertical temperature differences: in the actively warmed plots average temperatures at 150 cm above ground were 2.28 K (OTC-EH) to 2.46 K (OTC-WC) lower than at 10 cm height while they were only marginally lower (0.2 to 0.5 K) in the two control plots (Table 5). This demonstrates that both the electric heater and the warming cables caused pronounced warming near the ground but only moderate warming at 150 cm above ground. For the electric heater in OTC-EH this was due to the way it was installed, blowing the heated air toward the ground. Since the warming cables in OTC-WC were laid out on the ground surface, they mostly warmed the near-ground air and the top-soil, which has been demonstrated in other studies (e.g., Hagedorn et al., 2010).

Vertical heat exchange in both set-ups occurred mainly through natural convection, i.e., the buoyancy of warmer, lower density air. This effect may have been enhanced passively by the OTC walls during the day. However, the relatively large vertical within-chamber temperature differences suggest that internal air mixing in the actively warmed OTCs was limited. It is also unclear to what extent air escaped through the open tops of the OTCs. The escape of warm air may be prevented by covering the OTCs at least partly. This may, on the other hand, intercept precipitation depending on the specific design.

Horizontal temperature distribution in OTC-WC was similar to the two control plots, with small mean temperature gradients in horizontal direction between the OTC center and the OTC wall in the range of −0.09 to +0.24 K/m (Table 4), which may be explained by the layout of the warming cables that covered the complete radius of the OTC. In contrast, in OTC-EH, the heat source was concentrated in the center of the plot. To compensate for this technical constraint, we distributed the warm air more evenly over the chamber radius by installing small ventilators, which blew the warmed air in radial direction toward the OTC walls. A homogeneous temperature distribution was not completely achieved, though. The horizontal mean temperature gradient at 10 cm height in OTC-EH of 2.44 K/m was clearly higher than in the other plots (Table 4), underlining the difficulty to achieve a homogeneous temperature distribution within the OTC using this method. Temperature gradients in OTC-EH were smaller in experimental runs 4 and 5 than in runs 1–3 (1.78 K/m vs. 2.2 K/m), which shows that the 30° frustum added to the setup in runs 4 and 5 helped to achieve a more homogeneous temperature distribution in OTC-EH, which is also reflected in the MTSC values (Table 4). The temperature distribution in OTC-EH was clearly less homogenous in experimental run 6 compared to all other runs. This shows that the heating power of the electric heater only insufficiently produced homogenous warming when ventilators were blowing heated air in all directions as compared to only one half of the OTC in the other runs. It might be considered to force air mixing in large OTCs by installing larger or more effective fans. However, the increased air movement caused by these devices may exacerbate the potential side effects of ventilators, i.e., increased evapotranspiration and forced movements of the plants. Alternatively, we suggest to integrate the temperature gradient in OTC-EH in the experimental design. Such a design would allow to test different degrees of warming within a single OTC with strongest warming in the center of the OTC and decreasing degrees of warming toward the walls. A similar concept has been suggested for warming studies on rice paddies by combing an OTC with a solar-heated air-introduction tunnel (Chiba and Terao, 2014).

### Ecological Implications of Warming Methods

Climate manipulation experiments are valuable tools in global change ecology. While small passive OTCs can adequately warm low-stature plant communities or early life stages, larger-sized, actively warmed OTCs are required to study long-term warming effects on taller vegetation, such as tree saplings. Our large OTCs in combination with an electric air heater or warming cables as external heat source achieved a mean daytime warming of 2.5 to 2.9 K. This amount of warming approximately corresponds to recent IPCC climate change scenario predictions for the middle of the century (Stocker et al., 2013) and a similar amount of warming has frequently been shown to alter phenology, growth and survival of tundra plant communities in passive warming experiments (e.g., Arft et al., 1999; Elmendorf et al., 2012). Active warming experiments revealed similar responses in forest ecosystems, e.g., physiological acclimation (Drake et al., 2016) and phenological shifts at both ends of the growing season (Bronson et al., 2009; Gunderson et al., 2012; Richardson et al., 2018).

Since ecosystem responses to environmental changes are not always linear (Amthor et al., 2010), experimental designs incorporating environmental gradients are required to identify non-linear or threshold responses. Such a design has been implemented in the SPRUCE experiment with several large chambers that are designed to produce different levels of warming up to +12 K relative to ambient temperature (Richardson et al., 2018). As an alternative, we suggest to plan experiments such that the within-chamber temperature gradients that we observed in the actively warmed OTCs are incorporated in the experimental design for testing the non-linearity in plant and ecosystem responses to warming. Furthermore, the observed influence of the surrounding landscape on temperatures underlines the importance of a robust experimental design with replicate blocks to take such confounding effects into account.

The reported results were measured with empty OTCs in order to minimize biotic inferences with the heating setup and facilitate the understanding of physical effects occurring in the OTCs. We expect that plants in the OTCs will influence the warming depending on plant density, height and species identity. However, it is important to test the effect of the plants on warming with the actual plants before the start of an experiment. Since our experiments were run during fall and early winter, the reported results are conservative and higher degrees of warming are expected during spring and summer due to more sunshine hours and higher intensity of solar irradiance.

## Conclusion

Based on our findings for warming experiments with large OTCs we conclude that the temperature distribution in a passive OTC is reasonably homogeneous across space. The maximum amount of warming that can be achieved remains limited, though, and is not constant over time because the passive warming effect is small or inexistent during the night and under cloudy weather conditions. In contrast, the warming that can be achieved in actively warmed OTCs is substantially greater than in passively warmed chambers–provided that sufficient heating power is installed. Temporal variation in the achieved warming is much smaller than in a passive OTC. Nevertheless, the active warming methods have the disadvantage of producing uneven horizontal and vertical temperature distributions, although horizontal temperature variation is considerably smaller when using warming cables. The observed horizontal and vertical within-plot temperature variation can be several times larger than the target temperature difference in the study, which is a critical issue when these methods are used to study plant responses to warming such as changes in phenological or growth traits. Considering these findings, we emphasize the importance of a statistically robust experimental design and suggest to incorporate within-chamber temperature gradients in the experimental design instead of aiming to achieve a homogeneous temperature distribution within the OTCs. This approach requires to measure and report the spatial and temporal temperature distribution within the experimental chambers during the experiment (see also recommendations in Ettinger et al., 2019), and to consider the temperature variation in the interpretation of observed changes in plant growth and phenology.

## Data Availability Statement

The datasets generated for this study are available on request to the corresponding author.

## Author Contributions

ERF, LS, YV, TW, and BM conceived the ideas and designed the methodology. LS and ERF collected the data. ERF, BM, and LS analyzed the data. ERF and BM led the writing of the manuscript. All authors contributed critically to the drafts and gave final approval for publication.

## Conflict of Interest

The authors declare that the research was conducted in the absence of any commercial or financial relationships that could be construed as a potential conflict of interest.

## Footnotes

1 www.lwf.ch

2 www.icp-forests.net

## Supplementary Material

Supplementary Figure 1 Comparison of temperature readings of unprotected iButtons (red), iButtons protected with a commercial protective cover (yellow), and custom-fabricated radiation shields (orange) to the temperature recorded at the MODOEK climate station in approx. 1 m distance (black).

Supplementary Table 1 Materials and Equipment: List of required materials.
