# Supplementary figures and images for: Assessing the Effectiveness of in-situ Active Warming Combined With Open Top Chambers to Study Plant Responses to Climate Change
Source: Front Plant Sci. 2020 Nov 20;11:539584. doi: 10.3389/fpls.2020.539584 (PMC7714718; doi:10.3389/fpls.2020.539584)

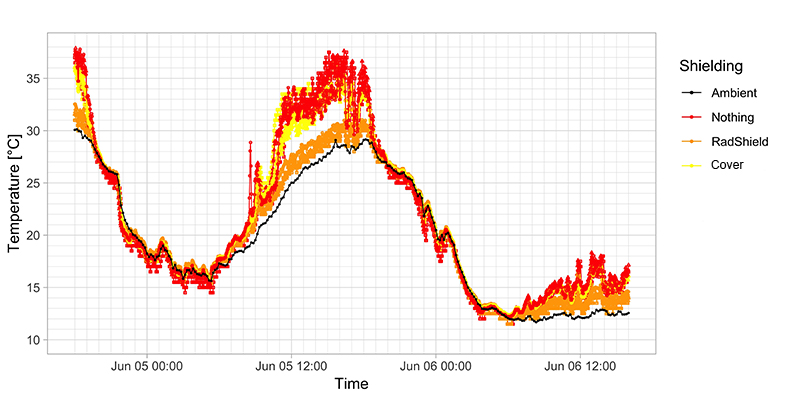

Supplement: Supplementary Figure 1 — Comparison of temperature readings of unprotected iButtons (red), iButtons protected with a commercial protective cover (yellow), and custom-fabricated radiation shields (orange) to the temperature recorded at the MODOEK climate station in approx. 1 m distance (black). [file Image_1.JPEG]
