# Supplementary material for: Assessing the Effectiveness of in-situ Active Warming Combined With Open Top Chambers to Study Plant Responses to Climate Change
Source: Front Plant Sci. 2020 Nov 20;11:539584. doi: 10.3389/fpls.2020.539584 (PMC7714718; doi:10.3389/fpls.2020.539584)
Supplement: Supplementary Table 1 — Materials and Equipment: List of required materials. [file Table_1.docx]

Assessing the effectiveness of in-situ active warming combined with open top chambers to study plant responses to climate change

**Running Title:** Effectiveness of in-situ warming methods

Esther R. Frei^1,*^, Luc Schnell^1,2^, Yann Vitasse^1^, Thomas Wohlgemuth^1^, and Barbara Moser^1^

^1^Swiss Federal Institute for Forest, Snow and Landscape Research WSL, Birmensdorf, Switzerland

^2^Department of Physics, ETH Zurich, Zurich, Switzerland

*Correspondence: Esther R. Frei, esther.frei@wsl.ch

**Keywords:** air temperature, electric heater (EH), phenology, relative humidity, spatial temperature distribution, warming cables.

Supplementary Material

**Table S1.** Materials and Equipment: List of required materials

| *OTC construction* | |
| --- | --- |
|  | |
| 18 | 2.5m roof battens (scaffolding for OTC) |
| 2 | 2 x 2.1 x 16 m Plastic foil (Lumisol clear AF, 200μ, UV Btransparent, Hortuna) |
| 1 | wood chips |
| 2 | 3 x 5.2 m soil layer separating plastic tissue |
| *OTC equipment (heat source devices)* | |
|  | |
| 1 | TROTEC TDE25 electric oven |
| 1 | 10 m extension cord |
| 1 | 1 wooden oven protection house (1.2 x 1.2 m plexiglas plate, 4 1.5 m wooden stakes) |
| 2 | CAMPLEX SOIL WARMING/HEATING CABLES 24.4 M 230/240 VOLTS, 300 watts |
| 1 | CAMPLEX SOIL WARMING/HEATING CABLE 48.8 M, 600 watts) |
| *Plot temperature measurement equipment* | |
|  | |
| 17 | iButton temperature loggers (DS1922L, DS1923) |
| 17 | self-made small radiation shields (type small RadShield after Terando et al. 2017) |
| 1 | 5.2 m string spanning diagonal through the 3 plots at 1.5 m height (where radiation shields with iButtons were attached to) |
| 1 | 5.2 m string spanning diagonal through the 3 plots at 0.10 m height (where radiation shields with iButtons were attached to) |
| 1 | 5.2 m string spanning diagonal through the 3 plots at 2.0 m height (where radiation shields with iButtons were attached to vertically) |
